# Allograft Versus Bioactive Glass (BG-S53P4) in Pediatric Benign Bone Lesions: A Randomized Clinical Trial

**DOI:** 10.2106/JBJS.22.00716

**Published:** 2023-01-19

**Authors:** Johanna Syvänen, Willy Serlo, Jenni Jalkanen, Ia Kohonen, Arimatias Raitio, Yrjänä Nietosvaara, Ilkka Helenius

**Affiliations:** 1 Department of Paediatric Surgery and Orthopaedics, Turku University Hospital, University of Turku, Turku, Finland; 2 Department of Children and Adolescents, Oulu University Hospital and PEDEGO Research Unit Oulu University and MRC Oulu, Oulu, Finland; 3 Department of Paediatric Surgery and Orthopaedics, Kuopio University Hospital, University of Kuopio, Kuopio, Finland; 4 Medical Imaging Centre of Southwest Finland, Turku University Hospital, Turku, Finland; 5 Department of Orthopaedics and Traumatology, Helsinki University Hospital, University of Helsinki, Helsinki, Finland

## Abstract

**Background:**

Benign bone cysts in children have a high risk of recurrence after bone grafting. The optimal treatment and filling material for these lesions are currently unknown.

**Methods:**

We compared cyst recurrence after intralesional curettage and filling with allograft versus bioactive glass (BG-S53P4; Bonalive) in a randomized clinical trial. The volume of recurrent cyst at 2-year follow-up was the primary outcome.

**Results:**

Of 64 eligible children, 51 (mean age, 11.1 years) were randomized to undergo filling of the cyst using morselized allograft (26) or bioactive glass (25). Twelve (46%) of the children in the allograft group and 10 (40%) in the bioactive glass group developed a recurrence (odds ratio [OR] for bioactive glass = 0.79, 95% confidence interval [CI] = 0.25 to 2.56, p = 0.77). The size of the recurrent cyst did not differ between the allograft group (mean, 3.3 mL; range, 0 to 13.2 mL) and the bioactive glass group (mean, 2.2 mL; range, 0 to 16.6 mL, p = 0.43). After adjusting for the type of lesion (aneurysmal bone cyst versus other), bioactive glass also did not prevent larger (>1 mL) recurrent cysts (adjusted OR = 0.42, 95% CI = 0.13 to 1.40, p = 0.16). The Musculoskeletal Tumor Society score improved significantly (p ≤ 0.013) from preoperatively to the 2-year follow-up in both groups (to 28.7 for bioactive glass and 29.1 for bone graft). Four (15%) of the children in the allograft group and 6 (24%) in the bioactive glass group required a reoperation during the follow-up (OR for bioactive glass = 1.74, 95% CI = 0.43 to 7.09, p = 0.50).

**Conclusions:**

Filling with bioactive glass and with allograft in the treatment of benign bone lesions provided comparable results in terms of recurrence and complications.

**Level of Evidence:**

Therapeutic Level I. See Instructions for Authors for a complete description of levels of evidence.

Benign bone lesions may weaken the structure of bone, resulting in pain and a risk of pathologic fracture^1^. The 2 most typical bone lesions during childhood are simple bone cysts (SBCs) and aneurysmal bone cysts (ABCs)^2-4^. These lesions tend to expand and thin the cortices of the growing bone^1,4,5^. Minimally invasive treatment using corticosteroid or polidocanol injections seldom results in healing of SBCs^6^, is associated with a high risk of recurrence in SBCs, and does not restore normal bone structure when used for SBCs and ABCs ^6,7^. The recurrence rate of SBCs is also high after curettage without bone grafting, equaling 19% (7 of 37) in the study by Aiba et al.^8^. Therefore, the standard treatment for larger, multicentric, or expanding tumors in weight-bearing areas in children has included bone grafting in an effort to decrease the risk of recurrence^2,9,10^. However, the reported local recurrence rate of ABCs has been relatively high (20%) even with implantation of allograft according to the study by Mankin et al.^2^. Risk factors for recurrence include young age, a location close to the growth plate, and an ABC rather than an SBC^2,11,12^. Measures to reduce the risk and size of recurrences have included use of a high-speed burr, phenol, sclerotherapy, cryotherapy, argon beam coagulation, and synthetic bone substitutes^13-18^.

Bioactive glasses are osteoconductive bone substitutes with osteoinductive, antimicrobial, and angiogenic properties^19^. Bioactive glasses release ions of calcium and phosphorus, resulting in biomineralization^19,20^. In addition, these ions and the slightly increased pH due to ion exchange reactions stimulate differentiation of mesenchymal stem cells, resulting in new bone formation. BG-S53P4 (53% SiO_2_, 23% Na_2_O, 20% CaO, and 4% P_2_O_5_) glass has been used for bone cavity filling in bone tumor surgery^10,19^. BG-S53P4 has been reported to be safe, well-tolerated, and suitable for lesion filling after curettage of a benign bone lesion, with good long-term results, in adults^19^. In 1 case report, treatment of a phalangeal ABC with BG-S53P4 in a 4-year-old girl did not disturb the growth of the bone^21^. Another study on pediatric ABCs also indicated that bioactive glass did not affect bone growth, and the recurrence rate of primary ABCs after surgical treatment (11%) appeared acceptable^15^. Furthermore, bioactive glass slowly dissolves, and the graft area remodels to bone over time. The slow dissolution rate might be beneficial, especially in the treatment of benign bone cysts, which are prone to recur in growing children^21^.

Autograft is the generally preferred material for benign bone lesions needing intralesional curettage and filling^2,12,19^. However, large cysts require a substantial amount of bone graft, which might not be available in younger children. Therefore, an optimal bone substitute would be useful. The aim of this study was to compare allograft and bioactive glass in the treatment of pediatric benign bone tumors with intralesional curettage. The primary outcome was the volume of recurrent cysts. We hypothesized that recurrent cysts would be smaller after filling with bioactive glass compared with allogeneic bone because the slowly dissolving glass would be more resistant to resorption by a recurrent tumor.

## Materials and Methods

The BEIBI (Bioactive glass vErsus allograft for BenIgn Bone lesIons) trial was a pragmatic multicenter randomized controlled trial comparing the use of bioactive glass and allograft as filling material after curettage of benign bone lesions in children. The study centers were 4 university hospitals in a Scandinavian country. The trial was registered at ClinicalTrials.gov (NCT04737590) and was approved by the Ethics Committees of the University Hospital (ETMK 53/180/2012). Written informed consent from patients or their caregivers was required for participation. This manuscript adheres to the CONSORT (Consolidated Standards of Reporting Trials) guidelines^22^.

### Patients

Patients between 4 and 16 years old who were scheduled for intralesional curettage and filling of a benign bone lesion were identified. Patients with a radiographically typical ABC or SBC in a load-bearing area or with a pathologic fracture were included, as well as those with other benign bone lesions in a load-bearing area. Pathologic fractures were treated with traction, a cast, or sling immobilization for a mean of 10 days (range, 7 to 21 days) before curettage, with no differences between the treatment groups. Patients with malignancy, bone marrow disease, a spinal lesion, or a lesion requiring preoperative biopsy before definitive intralesional management were excluded. Of the 51 patients (mean age, 11.1 years) included in the study, 26 were randomly assigned to the allograft group and 25, to the bioactive glass group.

### Interventions

The patients randomized to the allograft group received fresh-frozen morselized femoral head allograft and those randomized to the bioactive glass group received BG-S53P4 granules (Bonalive; Bonalive Biomaterials, Turku, Finland) as filling material at the end of the procedure. BG-S53P4 gained European approval for orthopaedic use as a bone graft substitute in 2006.

All procedures were performed by 5 experienced pediatric orthopaedic surgeons (3 to 20 procedures per surgeon). A cortical window, 15 × 20 mm in size, was made with an osteotome (Video 1). The tumor mass was curetted and sent for pathologic examination. Using a headlight for illumination, the contents of the cyst were removed systematically with curets and its lining membrane was removed using a high-speed burr. Phenol (5%) was applied to the cyst cavity with cotton swabs and neutralized with saline solution. The cyst volume was measured intraoperatively using saline solution. The cyst was then filled with morselized femoral head allograft or bioactive glass, depending on the randomization. The largest granule size, 2 to 3.15 mm, was used for ≥10-mL cavities in the femur, tibia, pelvis, and humerus. If the volume of the cavity was <10 mL, the intermediate granule size, 1 to 2 mm, was utilized. If the cyst was in the hand area, then the smallest granule size, 0.5 to 0.8 mm, was used. Intraoperative fluoroscopy was used to ensure that the entire cyst was filled properly. Locking-plate osteosynthesis was used in high-risk locations (such as proximal femur) to prevent a pathologic fracture.

### Follow-up Protocol

All patients were assessed preoperatively (baseline) and at 6 weeks and 3, 6, 12, and 24 months postoperatively. All follow-up visits included clinical examination, radiographs, and assessment of limb function using the Musculoskeletal Tumor Society (MSTS) score^23^ for the lower or upper extremity. Magnetic resonance imaging (MRI) was acquired preoperatively and at 3 months and 2 years postoperatively.

### Objectives

The objective of this pragmatic randomized clinical trial was to assess the rate and volume of recurrence in each study group. Our hypothesis was that the bioactive glass would reduce the size of the recurrent cyst, as it is a slowly resorbable bone substitute. We also hypothesized that the glass would not interfere with bone remodeling or cyst resolution.

### Outcomes

The primary outcome was the volume of the recurrence as measured on 2-year follow-up MRI. If the patient underwent a reoperation for recurrence before the 2-year follow-up, the size of the recurrence on the MRI prior to the reoperation was used as this end point. A musculoskeletal radiologist and a pediatric orthopaedic surgeon not involved in the treatment of these patients analyzed all images. Blinding of these evaluators was not possible, as the properties of allograft and bioactive glass on the images are different. The secondary outcomes were the recurrence rate, risk of reoperation (surgery or interventional radiology), MSTS score at the 2-year follow-up, and complications (blood loss of >50% of calculated blood volume, deep surgical site infection, pathologic fracture, and growth disturbance).

All patients had preoperative radiographs (anteroposterior and lateral) and MRI of the lesion, which included T1 and T2-weighted, STIR (short tau inversion recovery) or FS (fat saturation), and gadolinium-enhanced sequences. The recurrence of bone lesions was classified using the Neer classification^24^ modified to include the information provided by MRI^25^. Grade 1 represents complete evacuation and defect healing with no recurrence. Grade 2 represents successful defect healing with suspicion of static minor residua on MRI. Grade 3 represents tumor recurrence without a reoperation, and Grade 4 represents tumor recurrence needing a reoperation. Grades 3 and 4 were defined as recurrences and grouped together in the present study. The recurrence size was carefully measured on MRI using digital radiographic software. Growth disturbance was defined as an osseous bridge over the growth plate or development of a leg-length discrepancy or mechanical axis deviation during follow-up verified with radiographs or MRI.

### Sample Size

A study sample calculation using a study power of 80%, type-I error (alpha) of 0.05, and estimated effect size of 1.0 indicated that 16 patients per group were required. The effect size estimate was based on a retrospective study^15^ suggesting that the recurrent lesions in patients receiving bioactive glass would be 50% smaller than those in patients receiving allogeneic bone graft. The required sample size was increased by 16% to take into account the potential need to utilize nonparametric methods. Finally, to take into account potential loss to follow-up, a total of 50 patients were enrolled.

### Randomization

The patients were randomized 1:1 to the allograft or bioactive glass group during surgery, when curettage and use of local adjuvants had been completed, using sealed envelopes that had been prepared by a research coordinator using a computer-based sequence^26^. Given the nature of the intervention and the ages of the patients, blinding was not performed; however, our professional biostatistician performed the analyses without knowing the randomization group.

### Statistical Analysis

Statistical comparisons were performed according to the intention-to-treat principle. The baseline characteristics, MSTS score, and recurrence size were compared between the groups with the use of Mann-Whitney U tests. Chi-square or Fisher exact tests were used for comparisons of categorical variables. Kaplan-Meier survival estimates were used to compare the timing of recurrences between the study groups. Log-rank tests were used to compare the survival curves of the study groups. Multivariable analysis was performed using logistic regression. Significance was set at p = 0.05, and tests were 2-tailed. Analyses were performed with use of JMP Pro (version 16.1.0 for Windows; SAS Institute).

### Source of Funding

This study was supported by foundations, national state research funding, and a grant from industry to the Research Institute of Helsinki University Hospital. The funding was used only for researchers’ salaries, and the funders had no role in the analyses or writing of the manuscript.

## Results

**Fig. 1:**
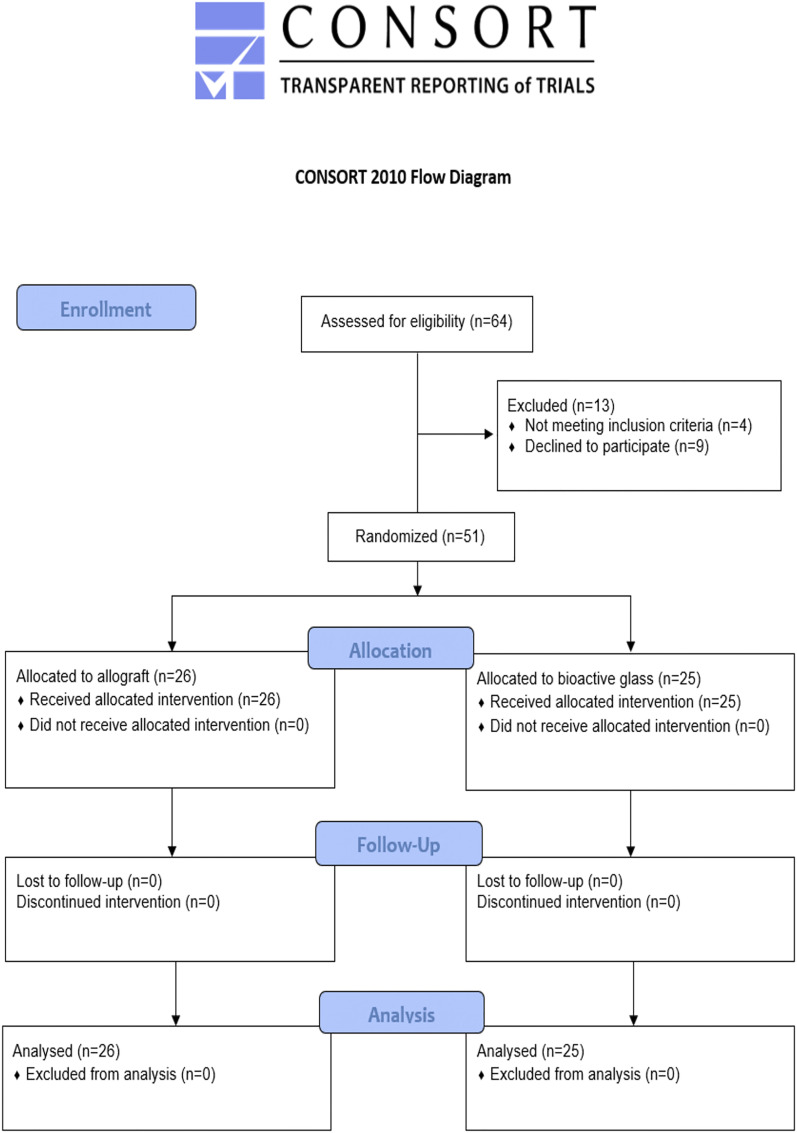
CONSORT flowchart. Twenty-five patients with complete follow-up (2 years) per group were required according to a sample size calculation, and a total of 55 tickets were randomized in sealed envelopes in advance to allow for potential loss to follow-up.

Sixty-four consecutive children aged between 4 and 16 years were screened (Fig. 1)^27^. Of these, 51 children (80%) and/or their parents consented to participate in the study between October 2012 and February 2019, and all patients had a minimum follow-up of 2 years (Table I). Following randomization, the allograft group consisted of 26 patients and the bioactive glass group, 25 patients. The allograft group included 14 patients with an SBC, 10 with an ABC, and 1 each with a nonossifying fibroma and fibrous dysplasia. The bioactive glass group included 14 patients with an ABC, 9 with an SBC, and 1 each with a nonossifying fibroma and an enchondroma. Sixteen (62%) of the children in the allograft group and 11 (44%) in the bioactive glass group were diagnosed with a pathologic fracture prior to the intervention.

**TABLE I:** Clinical Characteristics of the Study Groups

	Allograft (N = 26)	Bioactive Glass (N = 25)
Age at surgery* *(yr)*	11.1 (5.7-15.4)	11.2 (4.2-15.4)
Gender, M/F	24/2	14/11
Location		
Pelvis	0	1
Femur	5	5
Tibia	6	4
Fibula	1	1
Foot	4	6
Humerus	10	7
Hand	0	1
Type of lesion		
ABC	10	14
SBC	14	9
Nonossifying fibroma	1	1
Fibrous dysplasia	1	0
Enchondroma	0	1
Pathologic fracture *(no. [%])*	16 (62%)	11 (44%)
Surgical time* *(min)*	89 (37-260)	88 (45-245)
Intraoperative blood loss* *(mL)*	243 (0-800)	213 (5-1200)
Lesion size* *(mL)*	22.3 (1.5-80)	22.1 (0.4-110)

* The values are given as the mean, with the range in parentheses.

### Primary Outcome

**Fig. 2:**
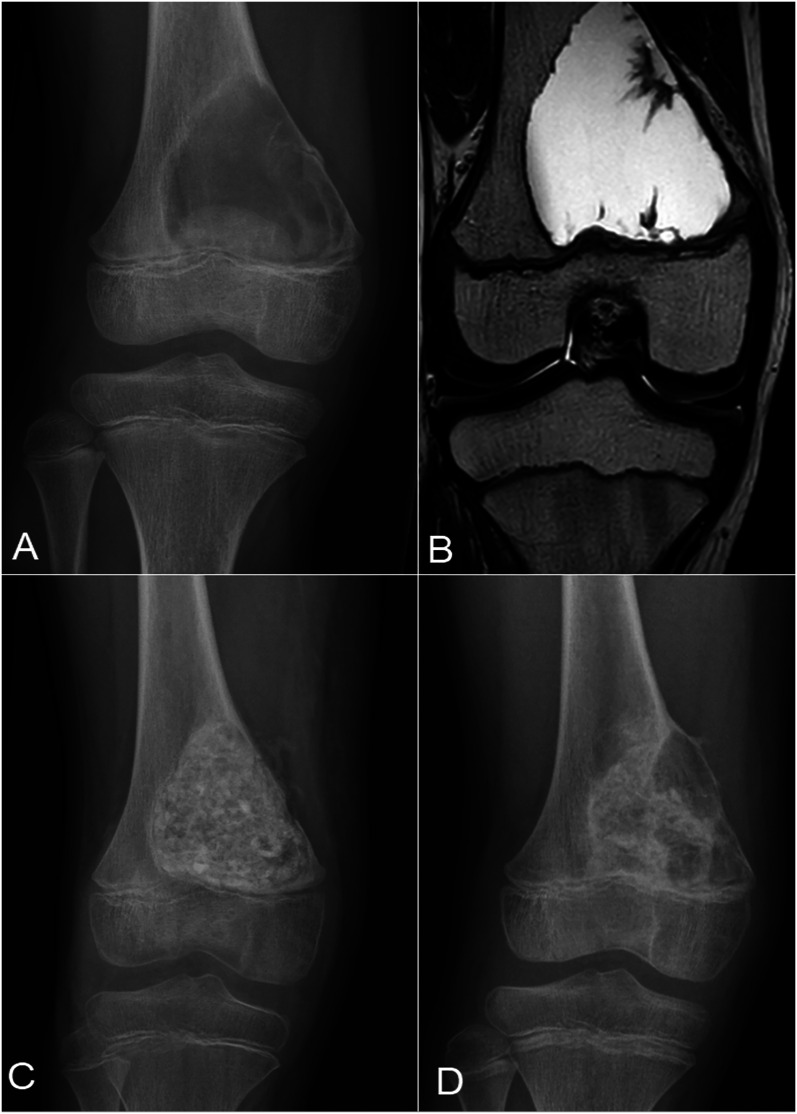
**Figs. 2-A through 2-D** An aneurysmal bone cyst in a 9-year-old boy. **Fig. 2-A** Preoperative radiograph. **Fig. 2-B** Preoperative T2-weighted MRI. **Fig. 2-C** Radiograph after intralesional curettage and filling of the defect using allograft. **Fig. 2-D** Radiograph showing a multiloculated recurrence at the 6-month follow-up.

**Fig. 3:**
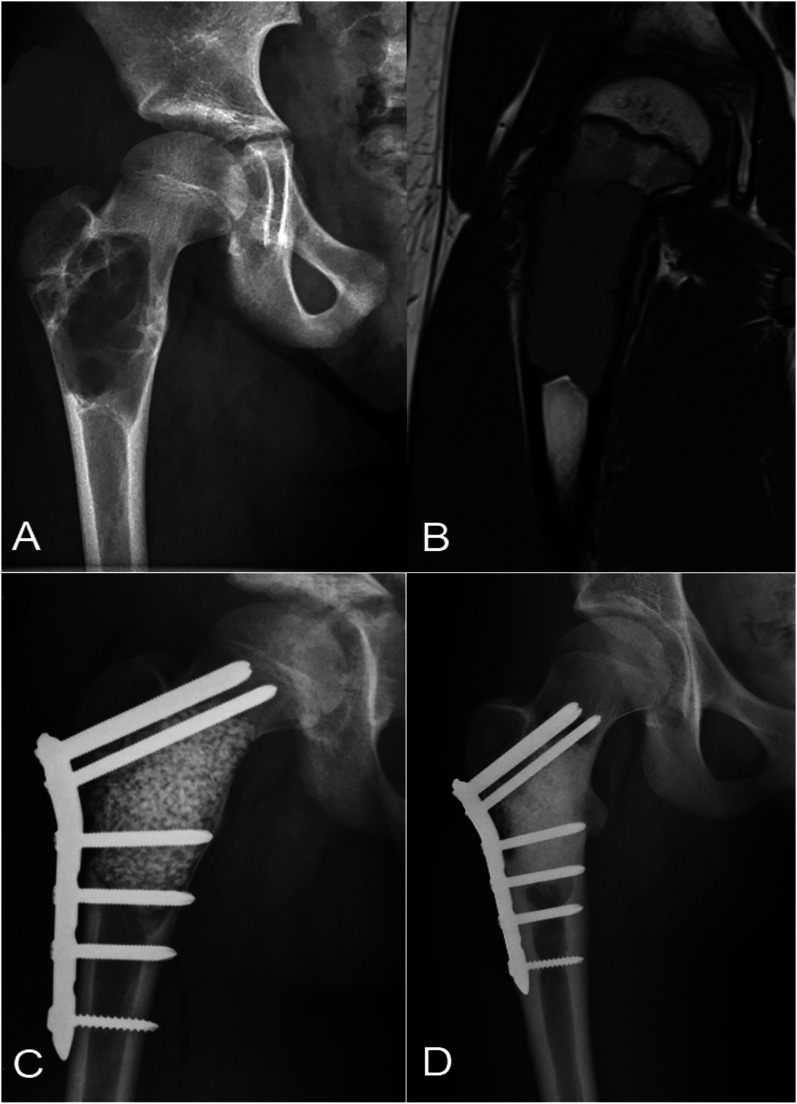
**Figs. 3-A through 3-D** An aneurysmal bone cyst in the proximal femur of an 11-year-old girl. **Fig. 3-A** Preoperative radiograph. **Fig. 3-B** Preoperative T1-weighted MRI. **Fig. 3-C** Radiograph after intralesional curettage, filling of the defect using bioactive glass, and locking-plate osteosynthesis. **Fig. 3-D** Radiograph showing a recurrence at the 2-year follow-up.

**Fig. 4:**
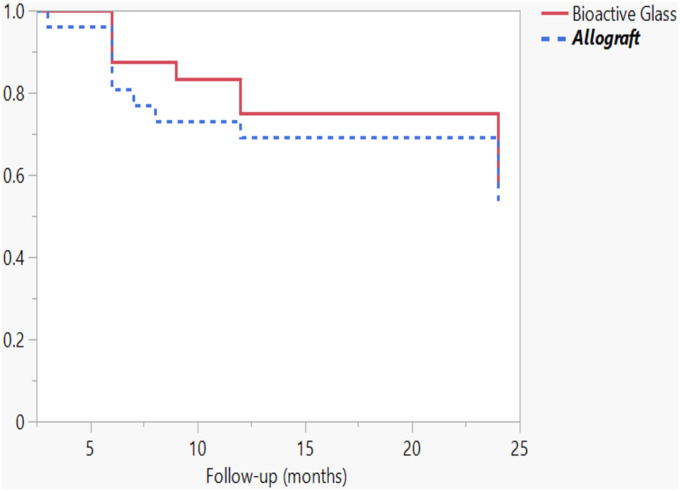
Kaplan-Meier survival curve for recurrence.

Twelve (46%) of the children in the allograft and 10 (40%) in the bioactive glass group developed a recurrence during the 2 years of follow-up (odds ratio [OR] for bioactive glass = 0.79, 95% confidence interval [CI] = 0.25 to 2.56, p = 0.77) (Figs. 2 and 3, Table II). The timing of the recurrences was similar, including for both early and late recurrences, according to the Kaplan-Meier curves (p = 0.69) (Fig. 4). The recurrence sizes did not differ significantly between the allograft (mean, 3.3 mL; range, 0 to 13.2 mL) and bioactive glass groups (mean, 2.2 mL; range, 0 to 16.6 mL) at the 2-year follow-up (p = 0.43). After adjusting for the type of lesion (ABC versus other), bioactive glass did not prevent larger (>1 mL) recurrent cysts at 2 years of follow-up (adjusted OR = 0.42, 95% CI = 0.13 to 1.40, p = 0.16).

**TABLE II:** Primary and Secondary Outcomes

	Allograft	Bioactive Glass	P Value
Recurrence of the lesion *(no. [%])*	12 (46%)	10 (40%)	0.66
Size of recurrence at 2-yr follow-up* *(mL)*	3.3 (0-13.2)	2.2 (0-16.6)	0.43
Reoperation *(no. [%])*	4 (15%)	6 (24%)	0.50
Hospital stay* *(days)*	2.4 (1-5)	2.9 (1-10)	0.66
MSTS score*			
Preoperative	23.4 (3-30)	22.4 (0-30)	0.91
6 weeks	23.3 (6-30)	22.7 (8-30)	0.67
3 months	27.0 (13-30)	27.4 (22-30)	0.41
6 months	28.5 (23-30)	29.0 (24-30)	0.20
12 months	28.7 (25-30)	27.9 (21-30)	0.50
24 months	29.1 (25-30)	28.7 (25-30)	0.51

* The values are given as the mean, with the range in parentheses.

### Secondary Outcomes

Four (15%) of the children in the allograft and 6 (24%) in the bioactive glass group required a reoperation (surgery or an interventional radiographic procedure) for a recurrence during the follow-up (OR for bioactive glass = 1.74, 95% CI = 0.43 to 7.09, p = 0.50). A special feature of the reoperations in the patients receiving bioactive glass was a uniformly very difficult approach to recurrent cysts with a high-speed burr through the extremely hard mixture of glass and newly formed bone. Thermal tissue injuries were prevented with continuous saline solution irrigation during this process, which did not result into pathologic fractures.

There were no growth disturbances (see Appendix), pathologic fractures, or deep surgical site infections. One (4%) of the patients in the bioactive glass group, who had a pelvic ABC, had intraoperative bleeding exceeding 50% of his blood volume. The MSTS score improved significantly from preoperatively to the 2-year follow-up in both the allograft group (from 23.4 to 29.1, p = 0.010) and bioactive glass group (from 22.4 to 28.7, p = 0.013), and the difference at 2 years was not significant (Table II).

### Subgroup Analysis

In an analysis of the subgroup of patients without a prior pathologic fracture, 14% (2) of 14 patients in the bioactive glass group and 50% (5) of 10 patients in the allograft group developed a recurrence (OR for allograft = 6.00, 95% CI = 0.86 to 42, p = 0.09). Similarly, in an analysis of the patients with a prior pathologic fracture, 73% (8) of 11 and 44% (7) of 16, respectively, developed a recurrence (OR for allograft = 0.29, 95% CI = 0.055 to 1.53, p = 0.24).

In a second subgroup analysis, the size of the recurrence did not differ between the study groups in the children with ABCs (mean recurrence size, 3.3 versus 2.2 mL, p = 0.43).

Video 1 Step-by-step video of the operative technique: Opening a cortical window to the cyst cavity using an osteotome, curettage, high-speed burring, tumor removal, phenol application, tumor volume measurement, filling the cavity with bioactive glass.

## Discussion

To our knowledge, the present study represents the first randomized study comparing filling materials for pediatric bone lesions. Benign bone lesions had a high risk of recurrence (averaging 43%) at 2 years after intralesional curettage and filling with either allograft or bioactive glass in the present study, with no differences in any outcome between groups.

Previous studies had suggested the risk of recurrence to be low after intralesional excision of benign bone tumors and filling with bioactive glass^4,15,19^. However, those studies investigated recurrences using radiographs. In the present study, MRI was used to assess recurrence twice. In contrast to our hypothesis, bioactive glass did not reduce the size of the recurrences, which were observed in 43% of all 51 children and in 40% of those in the bioactive glass group. A reoperation, either surgery or interventional radiography, was performed in one-fifth of the study cohort. A special feature of the reoperations in the patients treated with bioactive glass was a very difficult approach through the extremely hard glass and newly formed bone tissue.

### Limitations and Strengths

The present study included children needing intralesional curettage and cavity filling for a benign bone lesion. These were typically an SBC or ABC in a load-bearing area or with a pathologic fracture. Two patients had nonossifying fibroma, and 1 each had fibrous dysplasia and enchondroma. Nonossifying fibroma and fibrous dysplasia may have features similar to an ABC. A limitation of this study was the inclusion of multiple tumor types that differ in their intrinsic recurrence rates, which resulted in sample heterogeneity. Therefore, we performed a multivariable analysis that adjusted for the type of lesion as well as a subgroup analysis of the 24 patients with an ABC, which showed results similar to those in the overall study population. More than half of our patients (27 of 51) presented with a pathologic fracture before the intralesional tumor surgery, which was performed after a short delay in order to reduce intraoperative bleeding. The inclusion of patients with and without a pathologic fracture, and patients with and without internal fixation, increased the heterogeneity of the patient population further, representing a second limitation. However, pathological fractures and need for internal fixation are typical features of benign bone lesions and their management. A third limitation was the size of the cohort. Fourth, the reliability of the modified Neer classification^24,25^ has not been evaluated in pediatric bone lesions, and the MSTS score^23^ has been used in few studies of pediatric bone lesions^28^ and has not been validated in children. Fifth, there was potential for detection bias in the measurement of the lesion size and evaluation of the post-intervention imaging studies.

All 51 participating children reached the minimum 2-year follow-up. The curettage was performed in the same way in all patients irrespective of the type of the lesion. Researchers and families were not blinded to the treatment as bioactive glass and allograft have different imaging characteristics. Results were analyzed using the intention-to-treat principle. Our patients were consecutively treated at 4 academic medical centers. The treatment protocol was standardized. MRI was acquired twice to diagnose recurrent lesions.

The ideal filling material for bone cysts after curettage is theoretically autograft bone^10,19,25^. However, adequate volume of autograft bone is not always available, especially when treating large cysts in smaller children. Harvesting autograft also requires an additional procedure that results in pain at the harvest site. Therefore, autograft and bioactive glass may not be regarded as equivalent from a clinical or ethical perspective.

### Conclusions

The recurrence rate of benign bone lesions in skeletally immature patients treated with intralesional curettage is high. In the present small randomized trial, we could not identify a difference between bioactive glass and allograft as the filling material after curettage in terms of either the recurrence rate or the size of recurrent lesions. Bioactive glass represents an alternative filling material for pediatric benign bone lesions, despite its 2 unique negative aspects: nonphysiologic calcification on follow-up radiographs and difficult removal during revision surgery.

## Appendix

Supporting material provided by the authors is posted with the online version of this article as a data supplement at jbjs.org (http://links.lww.com/JBJS/H410).

## Data Availability

A **data-sharing statement** is provided with the online version of the article (http://links.lww.com/JBJS/H411).
